# Superior Properties and Biomedical Applications of Microorganism-Derived Fluorescent Quantum Dots

**DOI:** 10.3390/molecules25194486

**Published:** 2020-09-30

**Authors:** Mohamed Abdel-Salam, Basma Omran, Kathryn Whitehead, Kwang-Hyun Baek

**Affiliations:** 1 Analysis and Evaluation Department, Nanotechnology Research Center, Egyptian Petroleum Research Institute (EPRI), Nasr City, Cairo PO 11727, Egypt; dabdelsalam2008@epri.sci.eg; 2 Department of Biotechnology, Yeungnam University, Gyeongbuk, Gyeongsan 38541, Korea; obasma@ynu.ac.kr; 3 Department of Processes Design & Development, Egyptian Petroleum Research Institute (EPRI), Nasr City, Cairo PO 11727, Egypt; 4 Microbiology at Interfaces, Manchester Metropolitan University, Chester Street, Manchester M1 5GD, UK; k.a.whitehead@mmu.ac.uk

**Keywords:** nanobiotechnology, fluorescent quantum dots, microbiological synthesis, biomedical applications

## Abstract

Quantum dots (QDs) are fluorescent nanocrystals with superb photo-physical properties. Applications of QDs have been exponentially increased during the past decade. They can be employed in several disciplines, including biological, optical, biomedical, engineering, and energy applications. This review highlights the structural composition and distinctive features of QDs, such as resistance to photo-bleaching, wide range of excitations, and size-dependent light emission features. Physical and chemical preparation of QDs have prominent downsides, including high costs, regeneration of hazardous byproducts, and use of external noxious chemicals for capping and stabilization purposes. To eliminate the demerits of these methods, an emphasis on the latest progress of microbial synthesis of QDs by bacteria, yeast, and fungi is introduced. Some of the biomedical applications of QDs are overviewed as well, such as tumor and microRNA detection, drug delivery, photodynamic therapy, and microbial labeling. Challenges facing the microbial fabrication of QDs are discussed with the future prospects to fully maximize the yield of QDs by elucidating the key enzymes intermediating the nucleation and growth of QDs. Exploration of the distribution and mode of action of QDs is required to promote their biomedical applications.

## 1. Introduction

Semiconductor nanocrystals are innovative nano-sized materials with several beneficial properties. They are also designated as quantum rods (QRs), quantum dots (QDs), and quantum particles. QDs are defined as “almost rounded shaped nano-sized materials confined in a three-dimensional structure with a size ranging between 1–10 nm and they possess quantum properties due to Bohr radii” [1]. They are often called synthetic atoms, since they possess traits identical to that of normal atoms, such as being spatially localized in a 3D structure and possessing discrete energy levels [2]. QDs are named as such because they demonstrate a quantum confinement regime, which means that their electron wave function is related to particle size [3]. QDs were first discovered by the Russian physicist Alexey I. Ekimov in the early 1980s. Ekimov and Onushchenko investigated the quantum configuration of CuCl-immersed silicate in glass ceramics [4]. Since then, several investigations have been performed to discover a wide variety of QD-immersed glass ceramics [5,6,7]. Glass ceramics containing QDs have gained considerable notice because they can be successfully applied in optoelectronic devices, sensors, and photocatalysts [8]. The systematic development of QD science was motivated by Luis Brus, who derived a relationship between the size and band energy gap (BEG) of semiconductor nanoparticles [9]. The term “quantum dot” was used for the first time to characterize a three-dimensionally confined semiconductor quantum well by Brus et al. [10]. In 1993, Horset Weller was the first to identify the quantum confinement features of semiconductor QD particles [11]. QD absorption and emission spectra are usually size tunable. The difference in energy levels between both the activated and resting states of QDs is designated as the BEG of QDs. When QDs absorb fluorescent light, they become excited. The frequency or magnitude of absorbed light is related to both the BEG and the size of the QDs. To return to their original resting state, the QDs emit the same frequency of absorbed light. However, QD size is indirectly correlated to their BEG level (i.e., small-sized QDs release high-energy light, which is blue in color, whereas the large-sized QDs emit low-energy light, which is red in color) [12].

A new and reproducible method for synthesizing high-quality as well as monodispersed QDs was further developed in 1993 by Murray et al. [12]. In 1998, QDs were introduced for the first time as fluorophores owning distinctive features that make them more advantageous over conventional organic dye molecules [13]. Since that time, considerable progress has been observed to discover their potency in nanomedicine and nanobiotechnology as promising tools for use in diagnosis and therapies. It took nearly a decade since 1998 for new advances in QD research to allow the production of a successful preparation of colloidal cadmium-based chalcogenides (CdX), where X refers to sulfur (S), selenium (Se), or tellurium (Te) [14].

QDs are characterized by powerful light absorbance, symmetric and narrow emission bands, bright fluorescence with a broad range of excitation, high photo-stability, and slow excited-state decay rates [15]. QDs are usually synthesized via several top-down and bottom-up approaches. In a top-down approach, QDs are synthesized by thinning the bulk semiconductor material. Some top-down approaches employed to synthesize small-sized QDs involve reactive-ion etching, electron beam lithography, and wet chemical etching [1]. These techniques, however, retain impurities in the produced QDs, which lead to structural imperfections. Bottom-up production of QDs includes wet-chemical methods such as competitive reaction chemistry, micro-emulsion, sonic waves, microwaves, sol-gel, hot-solution decomposition, and electrochemistry [16].

Although the chemical synthesis of QDs opened up new avenues for preparing semiconductor nanocrystals with tunable shapes and sizes with desirable optical features, there are some disadvantages of the chemical preparation of QDs [17], including high energy requirements, release of hazardous organic and inorganic byproducts to the surrounding environment, and the high costs. Several organophosphorus solvents involved in the chemical synthesis of QDs can reach up to 90% of the total production costs [18]. Accordingly, the selection of solvents is an important issue affecting the exponential impacts of the chemical synthesis routes. Furthermore, instability has limited their potential applications, particularly in biomedical fields; therefore, more research is required to overcome such problems.

The implementation of eco-friendly, feasible, and biocompatible synthesis procedures is immensely desired. Synthesis of QDs by biological routes proposes cost-effective, low-toxic, and environmentally friendly alternatives to the chemical synthesis methodologies. Microorganisms are superior biological nano-factories which can be beneficial in synthesizing QDs. Biosynthesis of QDs has diverse of potent benefits which can be exploited particularly in biomedical applications. Microorganisms contain huge pools of diversity which confer them the inherent potential to mediate the synthesis of QDs. Microorganisms such as bacteria, yeast, and fungi are preferred for the synthesis of QDs due to several factors, including easy cultivation and their ability to grow at ambient conditions of pressure, temperature, and pH [19]. Additionally, due to their high adaptation and tolerating capacity to toxic metal containing environments, microorganisms possess unique intrinsic potency to mediate the synthesis of QDs by applying reduction mechanisms either extracellularly or intracellularly [20]. Microbial enzymes play the key role for transforming the precursor metal ions to their nanoparticle states.

The key advantage of bacterial-based synthesis of nanomaterials is their defense or resistance mechanism. Bacteria transform toxic metal ions to their nanoscale forms as a result of the stresses caused by the metal ions toward the bacterial cells [21]. Interaction takes place between the negatively charged bacterial cell wall and the positively charged metal ions. Nonetheless, certain restrictions occur such as the poor control over the sizes, geometries, and distributions of the prepared particles.

Fungi are more advantageous compared to bacteria in many ways involving: (i) fungal mycelia have the capacity to resist agitation, flow pressure, and many other harsh conditions in bioreactors; (ii) they are easy to handle; (iii) they can be easily monitored in downstream processing; (iv) out of microbes, they are the most potential candidates for large scale production because they secrete large amounts of enzymes leading to high formation of nanoparticles; and (v) they possess a very high cell wall binding capacity [22,23,24]. However, the use of fungi to mediate the mycosynthesis of nanoparticles is time-consuming and must be challenged in order to establish an economical method for up-scale production [25]. Moreover, problems such as slow reaction time, control over particle size and identification of the exact biochemical and molecular mechanisms for the microbial synthesis of QDs are among the research challenges that need extensive investigations.

An ideal fluorescent agent for biomedical applications has to possess the following criteria: (i) emissible of high fluorescence quantum yield; (ii) cyto-compatible; (iii) highly stable; (iv) reproducible; and (v) easily functionalized [13]. Traditional fluorescent probes or fluorophores used in biomedical applications involve organometallic and organic dyes such as rhodamines, fluoresceins, and cyanins. However, they have prominent defects such as broad emission spectra and narrow excitation spectra with discrete absorption bands [26]. In addition, they are susceptible to photo-bleaching, and the photochemical stability is usually poor with short fluorescence lifetime. Thermal stability and dispersibility are usually dye class-dependent. They also have limited usages particularly for multi-color detection because of spectral overlapping [27]. Fluorescence intensity and lifetime of organic dyes are influenced by viscosity, ionic strength, polarity, and pH [28]. The toxicity ranged from low to high is dye-dependent.

Contrary to traditional fluorescent probes, superb optical and physico-chemical properties are endowed to QDs because of their unique atomic configuration and particle size [15]. QDs have narrow emission spectra and broad excitation spectra with steady increasing bands [26]. They are resistant to photo-bleaching with high photo-chemical stability and also have a long-time fluorescence intensity. Thermal stability and dispersibility are dependent on the core–shell structure and ligand chemistry; QDs are ideal for multiplexing experiments because they generate high fluorescence quantum yield [29]. Toxicity can be due to the leaching of the heavy metal and inorganic element contents, therefore can be reduced by using specific biocompatible materials during the synthesis processes. Adopting suitable biocompatible materials helps to modify the QD surface, thereby minimizing the leaching of metal ions [30]. Additionally, reproducibility can be achieved by controlling the process parameters during the synthesis of QDs.

This review introduces a comprehensive outline of the basic structure of fluorescent QDs and their unique properties. The fabrication of QDs mediated by microbial machineries, e.g., bacteria, fungi, and yeast, is reviewed in depth. This review depicts the versatile biomedical applications of QDs as extremely promising tools, such as in disease detection, drug delivery, single-protein tracking, biosensors, and cellular labeling.

## 2. Structural Composition of QDs

**Figure 1:**
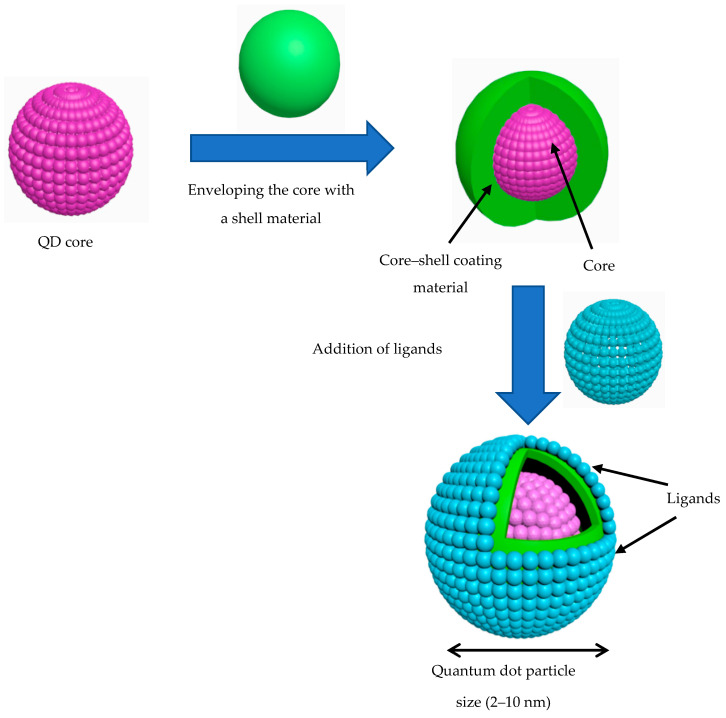
Quantum dot basic structure (core, shell, and ligands).

The quantum confinement regime of QDs usually occurs within 1–10 nm sized nanoparticles [31]. QDs structurally consist of a semiconductor core overlapped by an external shell, which is coated with ligands. The inorganic core structure controls the optical (e.g., light absorption and radiation) and semiconducting (e.g., electrical conductivity) properties. Ligands are key factors behind the development of QD properties, such as their colloidal solubility, stability, particle morphology, and particle size distribution (Figure 1) [32]. An ideal ligand should meet the following criteria: (i) provide the QDs with high stability and solubility in biological buffers; (ii) retain a high resistance toward photo-bleaching and other photophysical characteristics in aqueous media; (iii) contain functional groups to facilitate conjugation with biomolecules; and (iv) reduce the overall hydrodynamic size [33]. The most common ligands used include carboxylic acids (-COOH), alcohols (-OH), primary amines (-NH_2_), long-chain organophosphates, and thiols (-SH) [34]. The shell is mainly comprised of Type II–VI and IV–VI elements, which include configurations such as ZnO, ZnS, MgO, HgS, CdSe, and CdS [35]. The outer coatings play a vital role as physical barriers to protect the core from the surrounding medium. Representative examples of Type I core-shell materials involve (CdSe) InAs [36] as well as (CdSe) CdS and (ZnS) CdSe [37]. Inverse Type I core-shell materials are usually comprised of (CdS) CdSe [38], (CdS) HgS [39], and (ZnSe) CdSe [40]. QDs of Type II core-shell materials are primarily composed of (CdTe) CdSe [41] as well as (CdSe) ZnTe and (CdSe) ZnTe [42]. Single shell, multi-shell, and graded alloyed structure are the three major kinds of shell coatings [43]. The three forms of coatings vary mutually, according to the BEG and possible location of electrons′ energy state of both QD core and shell.

Electrons occur in a series of different energy levels, which are constant in bulk semiconductor material. At the nanoscale size, these rates become distinct because of the quantum confinement features. After a trigger, the electron hops from the valence to conduction level via the BEG, which leads to the formation of a hole behind. This hole has a positive charge. An exciton refers to the bound electron-hole pair in semiconductor materials and the effect of quantum confinement arises from the electrons that are physically confined in the 3D structure [44]. The exciton Bohr radius describes the mean physical space between electron-hole pairs, and this space is variable across different semiconductors. When the size of a semiconductor material resembles the exciton Bohr radius, its characteristics start to resemble QDs rather than the bulk semiconductor material. When electrons become excited to the conduction level, they return to their valence position, releasing electromagnetic radiation that differs from the initial stimulus. This frequency of emission is viewed as fluorescence and is size dependent on the BEG that can be modified by adjusting the QD size and surface chemistry [45].

## 3. Physicochemical Properties of QDs

**Figure 2:**
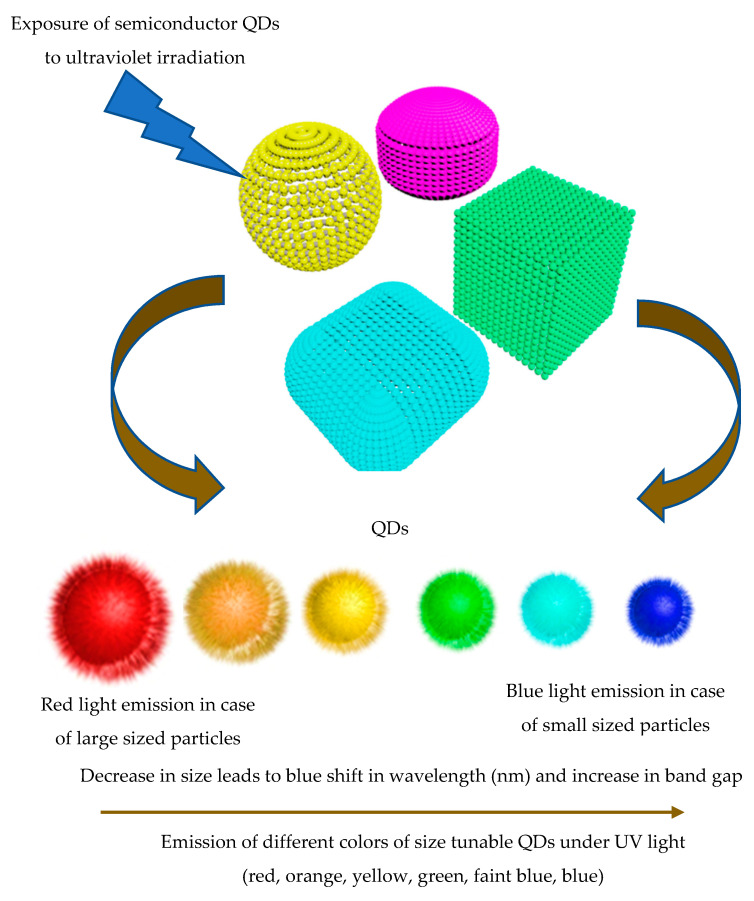
Quantum size confinement effect; irradiation of colloidal quantum dot (QD) particles under a UV light. Emission of different colors is dependent on the shape and size of the prepared particles.

QDs possess outstanding physio-chemical features as a result of the quantum confinement effect [46]. When material′s size is reduced, the quantum size features become prominent [47]. When the radius of nanoparticles is under the Bohr limit of excitation, the charge carrier′s Bohr radius becomes greater than that of the sphere. Thus, the charging carrier′s energy increases owing to containment within the sphere [48]. QD emission colors vary according to their size, chemical structure, and surface coating. The fluorescence of QDs can be adjusted over a broad wavelength (i.e., from 400 to 4000 nm). This facilitates measurements under the UV, visible, as well as near-infrared regions (NIRs) (Figure 2) [45]. Relatively large-sized QDs close to 10 nm in diameter emit red or orange emission at longer wavelengths with low-intensity radiation. In contrast, small-sized QDs close to 1 nm emit light at shorter wavelengths and possess green or blue emission with appropriate high levels of radiation intensity [35]. The emission wavelength of the core/shell CdSe/ZnS particles can be adjusted according to the particles′ size [49]. The emission wavelength changes from 480 (i.e., particle size: 2 nm) to 660 nm (i.e., particle size: 8 nm). Variations of wavelength emission of CdTe/CdSe QDs have also been shown to vary from 650 to 850 nm for 4 and 8 nm sized-QD particles, respectively [50].

### 3.1. Blinking

QD optical features are size tunable and are regulated by the quantum confinement effects since QDs are tolerant to photo-bleaching. Photo-bleaching refers to the procedure at which the luminescent materials decompose irretrievably because of optical excitation (or light-prompted response), which leads to a reduction in fluorescence strength [51]. Since QDs are resistant to photo and chemical degradation, they serve as potential candidates for use as imaging probes over long time periods [32]. Furthermore, QDs possess blinking behavior, which takes place throughout continuous molecular excitation. Blinking is thought to occur primarily due to the photo-induced carriers trapping and de-trapping, and therefore QDs oscillate between emitting and non-emitting levels. It is worth noting that such intensity differences on short periods are referred to as “quantum jumps”. Blinking takes place via two mechanisms: A-type and B-type blinking [52]. In A-type blinking, the presence of extra charges in the QDs leads to non-radiative decay rates. These charges appear due to the release of hole pairs photo-activated electrons. A temporal quenching of photoluminescence of a charged QDs occurs due to the recombination process between the excited hole pair electrons and the spare charges. However, no such correlation takes place in B-type blinking. To inhibit the blinking phenomenon in QDs, the charge carrier trapping process and trapping sites must be eliminated. Charge carrier trapping hinders charge transfer and recombination in QDs, hence limiting their efficiency [53]. A huge diversity of charge trapping sites has been speculated, experimentally measured, and analyzed via different theories. These states might be related to the surrounding ligands and medium. From the optical point of view, these states could be recognized in QDs via the incidence of broad and red-shifted emission peaks because of the rearrangement of the trapped charges. Accordingly, trapping minimizes the charge transfer in QD arrays. Further, withdrawing charges to a trap state reduces the overlapping of electron and hole wave functions inside the QDs and minimizes the recombination efficacy [53].

Several studies have demonstrated that, by increasing shell thickness, electronic insulation will take place, leading to the prevention of ionization [52]. Highly crystallographic structure of CdSe/CdS QDs with dense crystalline shells (i.e., made from CdS) have displayed a complete absence of blinking phenomenon, which was ascribed to the shell thickness [54]. Therefore, it was proposed that QDs that have a thick crystalline shell (e.g., ZnSe, CdS, and ZnS) do not blink [55]. Accordingly, the shell materials should be carefully chosen depending on the core of the prepared QD particles. It has been noted that tuning thicker shells made up of 4–6 monolayers offers more protection for the core material against degradation and photo-oxidation. This, in turn, reduces the lattice tightness between both the core and shell and increases the photophysical features of the QDs.

### 3.2. Stokes Shift

In the middle of the nineteenth century, and particularly in 1852, G.G. Stokes thoroughly investigated the fluorescence phenomenon and inspected how this phenomenon differs from that of incident light. In 1852, Stokes proposed his conclusions to London Royal Society [56]. His findings illustrated that there is one theory related to fluorescence namely, refrangibility of light (i.e., the degree of refraction is indirectly proportional to wavelength), which is shifted by dispersion to longer wavelengths [57]. This became known as Stokes′ law, which showed that fluorescence irradiation occurred at a higher wavelength than that of incident light. This law was accordingly named the Stokes shift in his honor. The Stokes shift is a significant feature of fluorophores that demonstrates the energy difference between both the absorption and emission states [58]. The Stokes shift is defined as “the variance between absorption and emission maximum bands” [59]. A large Stokes shift is commonly observed in semiconductor QDs and is an important feature used to identify their optical properties. Determining the Stokes shift is advantageous since, by using such criteria, fluorescence detection becomes easier, even at very low signal intensities [60]. The fluorophore is propelled to the excited levels (S_n_) after absorption, then quick relaxation takes place to the previous excited level (S_1_, internal conversion), and then returns to the original ground state (S_0_, fluorescence). The loss of excited energy during the internal conversion leads to red-shifted emissions (as opposed to absorption) and the incidence of Stoke shift. Small Stokes shifts (e.g., less than 50 nm) are observed in fluorescent proteins and organic dyes by photoluminescence spectroscopy, which cause an overlay between the absorption and emission spectra. Accordingly, the fluorophore absorbs the fluorescence emission in a process referred to as “self-absorption”. Self -absorption is defined as “a fluorescence quenching mechanism of which dramatically affects the optical features of fluorophores” [61]. Self-absorption causes problems, including aggregation-caused quenching and concentration quenching of fluorophores. This, in turn, can impede their applications in optoelectronic devices as well as their biological applications [62]. Therefore, several studies aimed to increase the Stokes shift of QDs. Among the applied strategies, doping of QDs [63], the use of alloy QDs [64], and the use of noble metal nanoclusters [65] have been utilized.

## 4. Microbial Synthesis of QDs

With the advent of nanobiotechnology, biological synthesis studies of nanomaterials managed to use “green chemistry” approaches, which are globally sustainable and economically viable. Microbial nanotechnology is a bio-driven scientific discipline that interconnects microbial biotechnology and nanotechnology. Numerous biological resources have been exploited for the biosynthesis of nanoparticles, including bacteria [66], fungi [67], algae [68], viruses [69], plant extracts [70], and agro-industrial wastes [71]. Fungi, yeast, and bacteria are currently receiving particular attention for the biological production of nanoparticles owing to their capacity to biotransform and bioaccumulate metals (Table 1). Among the investigated microorganisms, fungi are favored for the synthesis of QDs as they are efficient secretors of several biological molecules. Further advantages include the ease in biomass handling and economic viability. Nevertheless, bacterial-mediated synthesis of nanoparticles confers advantages since bacteria can be genetically manipulated for the expression of particular enzymes involved during the synthesis of metallic nanoparticles [72].

### 4.1. Mechanisms of Microbial Synthesis of QDs

**Figure 3:**
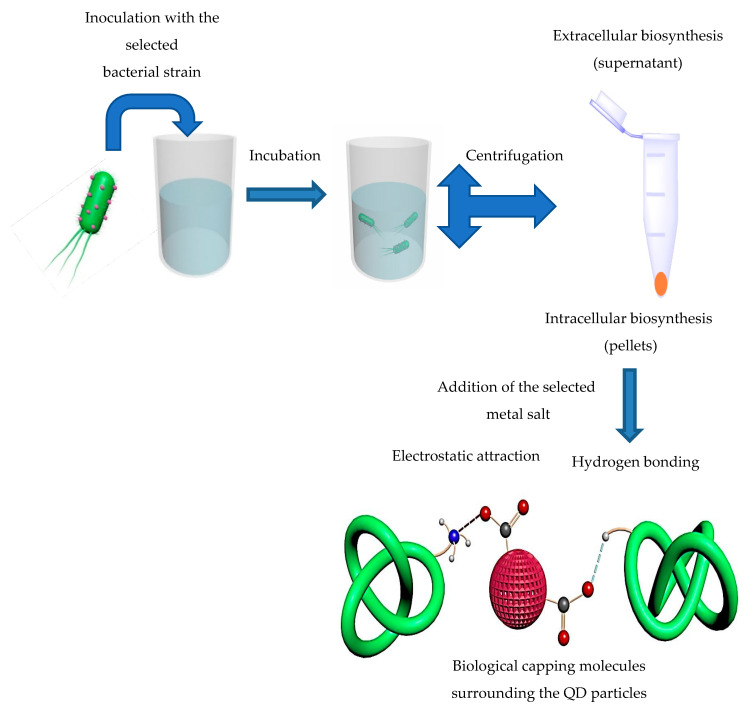
Extracellular and intracellular synthesis mechanisms of QD particles using bacteria.

Developing facile, low cost, and measurable methodologies for preparing QDs with controlled structures and distinctive properties represents a major challenge. Generally, two different mechanisms are applied: intracellular and extracellular syntheses mechanisms (Figure 3).

#### 4.1.1. Intracellular Microbial Synthesis of QDs

The intracellular microbial synthesis of QDs involves the penetration of dissolved ions to microbial cell cytoplasm via the manganese or magnesium transferring systems. When transported into the cell cytoplasm, the metal ions are processed into nanoparticles using intracellular enzymes [96]. When metallic nanoparticles are synthesized intracellularly, it gets difficult to handle downstream processes and the cost of recovering the nanoparticles may increase. To recover the QDs, the genetically engineered cells may be lysed, centrifuged, freeze-thawed, and purified through columns of anion exchange [97]. Therefore, alternate pathways for QD biosynthesis need to be investigated, which can overcome subsequent procedures required for QD extraction and purification. This is extremely crucial for designing reliable routes for the development of QDs on a large-scale utilizing microbial community.

#### 4.1.2. Extracellular Microbial Synthesis of QDs

To monitor the downstream procedures needed for QD intracellular microbial synthesis, one-pot extracellular manufacturing of QDs in microorganisms was developed. The microbial extracellular synthesis is rapid, scalable, and can be carried out under ambient conditions. The extracellular microbial synthesis of QDs is carried out with the help of the enzymes that reside in the cellular membrane or deposited in the growth medium [98]. The developed QDs are therefore either adsorbed on the cellular membrane or deposited in the growth medium. For instance, the extracellular biosynthesis of CdS QDs via the fungus *Fusarium oxysporum* was reported by Ahmad et al. [99]. Microorganisms produce specific enzymes such as reductases as a defense strategy. Such reductase enzymes could be involved during the QD extracellular biosynthesis [100]. The sections below introduce in depth overview of the synthesis of different QD particles via microbial machineries.

### 4.2. Different Types of Microbially Fabricated QDs

#### 4.2.1. Bacterial-Mediated Synthesis of QDs

*Escherichia coli* ATCC 29,181 is an ideal bio-factory to microbially fabricate CdTe QDs extracellularly [100]. CdTe QDs are synthesized from Cd and Te precursor metal salts via one-pot reaction using *E. coli*. Uniform spherical-shaped QDs with a size of approximately 2–3 nm were successfully observed by transmission electron microscope (TEM). X-ray diffraction (XRD) pattern demonstrated a sharp peak at 27.5 corresponding to the (111) planes, which indicated the cubic structure of CdTe. Since *E. coli* releases a large amount of proteins, especially metal-binding proteins, such proteins aid in the formation of the capping layer surrounding CdTe QDs. The hydrodynamic size of the *E. coli*-derived CdTe QDs was found to be larger compared to that of the hydrothermally-prepared CdTe QDs. This suggested the presence of capping/coating layers surrounding the microbially-produced CdTe QDs. Zeta potential measurements recorded −36.4 and −19.1 mV for *E. coli* and the hydrothermally fabricated CdTe QDs, respectively. The difference in zeta potentials significantly indicated the presence of several coating materials surrounding the prepared CdTe QDs. Further, the highly negative zeta potential of *E. coli*-mediated CdTe QDs revealed the high stability of the biosynthesized crystals. Fourier transform infrared (FTIR) spectroscopy was used to detect the possible chemical configuration of the surface of the microbially synthesized CdTe QDs. Two absorption peaks were centered at 1668 and 1545 cm^−1^ that could be attributed to the amide I and II group bendings, respectively. Coating of the QDs with biomaterials following their production is considered an effective methodology to modify them in order to diminish their nanotoxicity. The prepared CdTe QDs were used to image cultivated cervical cancer cells in vitro after functionalization with folic acid, and they proved to be an effective substitute or complementary tool for using traditional organic staining methods.

Cadmium sulfide (CdS) QDs are typical sulfide nanoparticles that can be synthesized by microorganisms. An easy route for synthesizing CdS QDs by the photosynthetic bacterium *Rhodopseudomonas palustris* was proposed by Bai et al. [96]. The CdSO_4_ solution reacted with *R. palustris* biomass. After 48 h, the color of the reaction mixture changed to yellow, implying the synthesis of CdS QDs. A maximum absorption peak occurred at 425 nm, which was distinctive for quantum sized CdS particles. Moreover, XRD confirmed the crystallinity of the purified CdS QDs. TEM images showed a uniform distribution of CdS QDs with an average diameter of 0.25 ± 8.01 nm. The acidophilic bacterium of the *Acidithiobacillus* genus mediated the synthesis of CdS QDs [101]. Three *Acidithiobacillus* species, namely *A. thiooxidans*, *A. caldus*, and *A. ferrooxidans*, were exposed to sub-lethal concentrations of Cd^2+^ in presence of glutathione and cysteine. Red fluorescence was emitted by the reaction of *Acidithiobacillus* species with cadmium ions. The fluorescence of cadmium-exposed cells changed from green to red. The obtained CdS QDs displayed an absorbance band at 360 nm, as revealed by UV/Vis spectrophotometer. Once excited at 370 nm, broad emission spectrum appeared between 450 and 650 nm, which was distinctive to CdS QDs. Interestingly, microbially-fabricated QDs by acidophilic bacteria could withstand an acidic pH. These findings represent the first study to generate QDs with desired traits via extremophilic bacteria.

*E. coli* was tested as a bio-matrix to mediate the synthesis of CdS QDs by Yan et al. [79]. A sharp fluorescence emission peak appeared at 470 nm, indicating the presence of CdS QDs. Fluorescence inverted microscopy and TEM confirmed the presence of spherical shaped CdS QDs with a homogenous size of approximately 10 nm. The edges of the particles were lighter than the particles′ center, signifying that the biosynthesized QDs might be enclosed by an enveloping capping material. Energy dispersive X-ray spectroscopy (EDX) identified the elemental structure of the particles, which were mainly made of sulfur and cadmium. XRD mapping indicated the presence of a cubic crystalline construction of CdS QDs. This microbial synthetic route resulted in the formation of uniform sized particles of CdS QDs with great fluorescence intensity. This synthesis approach had several advantages, including moderate temperature, low toxic effects, and high efficacy.

Tellurite and tellurate are the most abundant forms of Te in nature [102]. The possibility of changing them into the less toxic elemental Te (Te^°^) was investigated by Forootanfar et al. [103]. A bacterial strain was identified as *Pseudomonas pseudoalcaligenes* and was isolated from a hot spring. *P. pseudoalcaligenes* was capable of synthesizing tellurium nanorods (Te QNRs) [103]. An absorption peak was demonstrated at 210 nm and EDX of the purified Te QNRs displayed a characteristic Te elemental peak at 3.72 keV. Cu peaks were also detected but they were derived from the TEM copper grid. The cytotoxic effects of the biosynthesized Te QNRs on different cancer cell lines A549, HT1080, HepG2, and MCF-7 were evaluated, and the 3-(4,5-dimethylthiazol-2-yl)-2,5-diphenyltetrazolium bromide (MTT) assay was employed. The results show a direct relationship between the Te QNR applied doses and the cytotoxic effect. A low toxic effect was conducted by the rod-shaped microbially-synthesized tellurium nanostructures compared with that of Te^4+^ ions.

#### 4.2.2. Fungal-Mediated Synthesis of QDs

*F. oxysporum* effectively mediated the preparation of highly fluorescent CdTe QDs at ambient conditions, as proposed by Syed and Ahmad [87]. CdTe QDs were successfully synthesized by reacting cadmium chloride and tellurium chloride with *F. oxysporum* mycelial biomass (20 g wet mycelia) for 96 h at 200 rpm. The fluorescence measurements were investigated via excitation of the reaction mixture at 400 nm. The mycosynthesized QDs demonstrated a fluorescence emission peak positioned at 475 nm, which was similar to chemically synthesized QDs. The XRD analysis showed intense peaks corresponding to (111), (220), and (311) planes. An energy dispersive X-ray (EDX) spectrum revealed signals corresponding to Cd, Te, O, and C. The C and O signals were likely due to X-ray emissions from proteins/enzymes present on the nanoparticle surfaces. X-ray photoelectron spectroscopy (XPS) illustrated the existence of Cd, Te, O, and C as the main elements. Thermogravimetric analysis (TGA) was carried out to identify the thermal properties (desorption/decomposition) of the as-prepared QDs. The TGA spectrum showed weight loss (30%) in the temperature range of 200–250 °C. The first weight loss was ascribed to the released biomolecules and water vapor, which coated the surface of the mycosynthesized QDs. Further degradation upon increasing the temperature occurred at the range of 500–700 °C. The prepared QDs exhibited antibacterial potential toward Gram-positive bacteria (e.g., *Staphylococcus aureus* NCIM 2079 and *Bacillus subtilis* NCIM 2063) and Gram-negative bacteria (e.g., *E. coli* NCIM 2065 and *Pseudomonas aeruginosa* NCIM 2200).

Selenium and lead-tolerable marine isolate *Aspergillus terreus* was used to mediate the mycosynthesis of fluorescent lead selenide QRs (PbSe QRs) [72]. An absorbance peak occurred at 872 nm, which was distinctive to quantum sized PbSe particles. A weak absorption peak at 375 nm appeared, indicating the presence of protein-capping agents. FTIR spectroscopy was used in the determination of the possible functional groups of the ligands capping the edges of the prepared QRs. The UV/Vis spectrum of the mycosynthesized PbSe QRs showed absorbance peaks at wavenumbers correlated with the presence of carboxylic and amide functional groups. Scanning electron microscope (SEM) images illustrated the presence of biogenic PbSe nanorods. The PbSe QRs crystallite size of 3.057 nm was predicted using the Scherer equation.

The phytopathogenic fungus, *Helminthosporium solani*, was incubated with an aqueous solution of cadmium chloride (CdCl_2_) and selenium tetrachloride (SeCl_4_) under ambient conditions. CdSe QDs were synthesized extracellularly. This was reported for the first time by Suresh [104]. The synthesized particles had 1% quantum yield and broad photoluminescence. An absorption peak at 350 nm was observed using a UV/Vis spectrophotometer, suggesting the formation of CdSe QDs. Absorption bands between 270 and 280 nm were also detected, and may have originated from proteins or peptides, thus demonstrating the possible involvement of protein or a peptide material as capping molecules. The photoluminescence properties of the mycosynthesized CdSe QDs were investigated by fluorescence spectral measurements following excitation at 380 nm. An emission band was observed at 430 nm. EDX depicted strong elemental signals for both cadmium and selenide. Particles were monodispersed, spherical in shape with an average diameter of 5.5 ± 2 nm, and a few square-shaped particles were also identified through the TEM images. The prepared particles were not in direct contact even within the aggregates, indicating powerful stabilization by the capping proteins/peptides. Particle size analysis showed that the particles had a mean diameter of 5.5 ± 2 nm. XPS was used to further validate the synthesis and assess sample purity and composition of biogenic CdSe QDs. The prominence of Cd and Se confirmed the synthesis and purification of the prepared QD particles.

The extracellular mycosynthesis of CdS QDs using the white rot fungus *Phanerochaete chrysosporium* was conducted by Chen et al. [88]. *P. chrysosporium* was incubated in cadmium nitrate tetrahydrate containing solution. The reaction solution turned yellow after 12 h, signifying the biogenic synthesis of CdS QDs. A maximum absorption peak occurred between 296 and 298 nm that was characteristic of the CdS quantum particles. The as-prepared CdS QDs emitted blue fluorescence at 458 nm. XRD confirmed the synthesis of crystalline CdS QDs with a face-centered cubic configuration. The average size of the particles was almost 2.56 nm, as calculated by the Scherer equation. TEM showed that the prepared particles were uniform in size. It was found that cysteine and proteins played a significant role during the formation and stabilization of the prepared CdS QDs.

*Rhizopus stolonifer* was used for the biomimetic synthesis of cadmium chalcogenide QDs [90]. The suspensions of CdTe and CdS QDs exhibited purple and greenish-blue luminescence, respectively, upon illumination via an 8 W UV lamp. Suspensions of both types of QDs remained highly stable even after four months of storage and did not exhibit any kind of aggregation. The average calculated crystallite sizes of both CdTe and CdS QDs were 7.6 and 8.8 nm, respectively, according to Scherer equation. XRD data concluded the cubic and hexagonal crystalline phases of CdTe and CdS QDs, respectively. More than 80% of the cells were viable, as confirmed by the MTT assay, using 20 μL of CdTe/CdS QDs. By loading the CdTe/CdS QDs in human breast adenocarcinoma (MCF-7) cell lines, better image contrast was obtained. These findings show that CdTe/CdS QDs could serve as alternatives to traditional organic dyes.

Increased demand of CdS QDs has led to the search for new synthesis methodologies to guarantee high production, precise control over particle size, and improved environmental friendliness within the production process, since several techniques use toxic solvents and consume high energy. In this context, Cárdenas et al. [92] used *F. oxysporum* for the preparation of hydrophilic CdS QDs. The mycelia of *F. oxysporum* were incubated with 1 mM of cadmium nitrate for 24 h at 30 °C. The biomass was then filtered. The filtrate became yellowish in color, depicting the synthesis of extracellular CdS QDs. This was confirmed by UV/Vis spectrophotometry, which revealed a characteristic band at 450 nm. Biosynthesized QDs were circular in shape with a diameter of 2.111 ± 6.116 nm and had a wurtzite crystalline structure. EDX analysis of the prepared CdS QDs showed the presence of Cd and S elements. TEM micrographs demonstrated the formation of circular-shaped agglomerated CdS QDs.

#### 4.2.3. Yeast-Mediated Synthesis of QDs

Among the most important QDs, CdTe QDs have been extensively investigated in biomedical and industrial applications due to their distinctive properties, such as high photo-stability, controlled and narrow emission spectra, and high quantum yield, compared to conventional fluorescent dyes. CdTe QDs are promising candidates in the biological imaging of living cells. CdTe QDs with tunable fluorescence emission spectra were effectively biosynthesized using yeast cells, as demonstrated by Bao et al. [105]. This was carried out via the incubation of a type of *Saccharomyces cerevisiae*, using Cd and Te metal salt precursors. The as-prepared CdTe QDs displayed size-tunable dependent emission spectra ranging from 490 to 560 nm. A high quantum yield of ~33% was obtained. The microbially fabricated CdTe QDs were naturally surrounded with proteins and showed exceptional biocompatibility and stability. TEM revealed the presence of well-dispersed CdTe QDs with a diameter ranging between 2.0 and 3.6 nm. XRD patterns of the biosynthesized CdTe QDs showed a diffraction peak centered at 2*θ*~26.7°, which corresponded to the (200) reflection for cubic CdTe QDs. The possible ligands that might have capped the as-prepared CdTe QDs were detected via FTIR spectroscopy. Two absorption peaks occurred at 1650 and 1566 cm^−1^, which corresponded to amide I and II functional groups, respectively. The molecular mass and chemical composition of the capping proteins were further analyzed by high performance liquid chromatography (HPLC). Interestingly, HPLC revealed the presence of two types of proteins with molecular masses of 7.7 and 692 kDa with percentages of 93.4% and 6.6%, respectively. These proteins were produced from the tested yeast cell under investigation and aided in the stability and the inhibition of any possible aggregation of the prepared CdTe QDs. Accordingly, the protein-capped CdTe QDs could be tremendously beneficial in bio-labeling and -imaging applications.

ZnS QDs are among the most important and attractive semiconductor nanoparticles particularly in infrared and fast switching optical devices. Mala and Rose [83] demonstrated the microbial synthesis of ZnS QDs by *S. cerevisiae* MTCC 2918. A characteristic surface plasmon resonance (SPR) band of ZnS QDs was detected at 302.57 nm. XRD pattern showed that the nanoparticles were in the sphalerite phase. Two photoluminescence spectra were revealed at 280 and 325 nm. This suggested that the yeast had inherent sulfate-metabolizing systems that, in turn, were capable of assimilating sulfate.

## 5. Biomedical Applications of QDs

**Figure 4:**
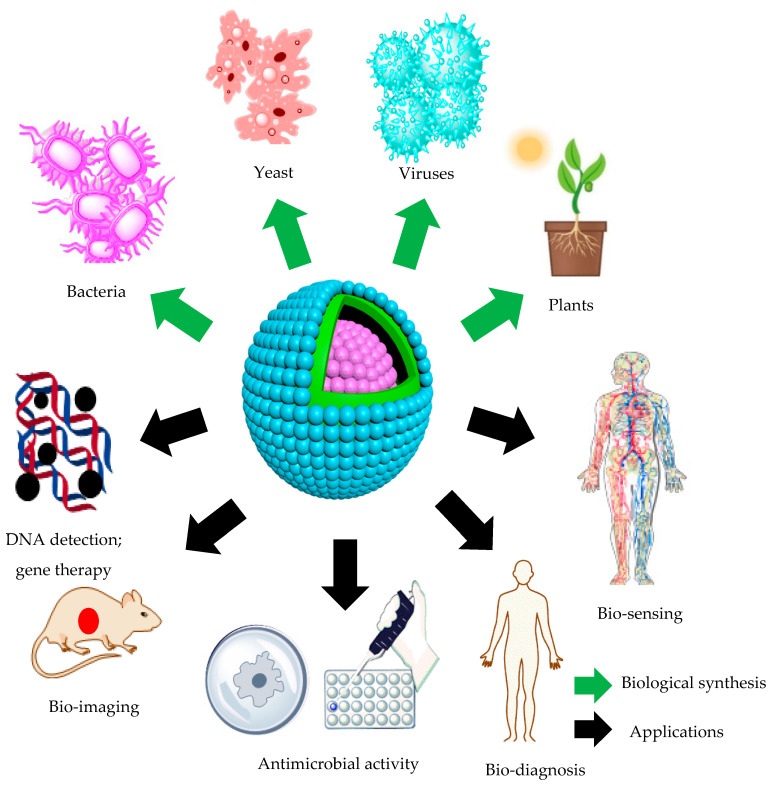
Scheme representing the different biological entities used for the biological fabrication of quantum dots (QDs) and their various applications.

Semiconductor QDs are used in several applications, ranging from optoelectronic to bio-molecular, as summarized in Figure 4. They are extremely promising nanomaterials that can be applied to bio-sensing, bio-imaging, DNA detection, telecommunication, lasers, photo-detectors, and photovoltaic devices [106]. QDs are promising candidates for bio-sensing owing to their exceptional physical, chemical, and optical traits and the ability to bind different biomolecules to their surface. Innovative applications of QDs developed the current techniques for proteins and DNA detection. Moreover, QDs are beneficial in immunochemistry via molecular tracking. They represent better alternatives for the fluorescent beads that are mainly employed for studying the dynamics of neurotransmitter receptors because of their very small size (approximately 1–10 nm) compared to latex beads (approximately 500 nm) [107]. The QDs also function as biological luminescent markers capable of recognizing molecular structures. Effective multicolor cell labeling with QDs can typically be achieved through receptor-mediated diffusion or unspecific endocytosis, which provokes primitive cellular mechanisms to transfer nanoparticles via the cell membrane. Endocytosis is henceforth the least destructive mode of delivery compared with traditional approaches, such as microcapillary injection or electroporation. Endocytosis depends on injecting the target material into the cell. The inserted material becomes surrounded by a part of the cell membrane, which then buds off from the main cellular body and forms a vesicle inside the cell.

The most commonly used fluorophores are organic fluorophores, such as genetically engineered fluorescent proteins and chemically manufactured fluorescent dyes. There are two noteworthy restrictions for using these organic fluorophores in different applications, as demonstrated by Drbohlavova et al. [108]. First, they cannot consistently fluoresce over long periods of time. Second, because of their quite broad emission spectra, they are not suited for multi-color bio-imaging applications. Accordingly, a suitable fluorescent marker should be biocompatible, highly fluorescent, and resistant to photo-bleaching.

In the late 1990s, Bruchez et al. [13] and Chan and Nie [109] published the first trials for the usage of QDs in imaging. Since then, researchers have exploited the properties of the QDs, in particular their photo-stability, luminosity, size-tunable optical and electronic features, and multiplexing potential, all of which enable QDs to be applied in a wide variety of different fields. Silver and its alloys were extensively employed for a number of years in a range of applications, including jewelry, coins, casting, and explosives [110]. Silver has also been demonstrated to have potent anti-microbial activity against various types of microbes, including bacteria, yeast, fungi, and viruses. During the last few decades, major scientific leaps in nanoscience have resulted in the development of quantum silver particles, which have biomedical potential as a result of their photoluminescence in the NIR. The use of such QDs may assist in certain biomedical applications such as photodynamic diagnostics, therapy, and in vitro imaging [111].

The silver-based QDs with potential use in biomedical applications are silver chalcogenide QDs (Ag_2_X, where X = Te, Se, or S) as they exert low toxic effects in comparison with the other conventional QDs, such as CdSe, CdTe, and CdS [112]. Silver chalcogenide-based QDs also have a narrow band gap. For instance, the EBG of Ag_2_S, Ag_2_Se, and Ag_2_Te are in the order 0.9–1.1, 0.15, and 0.67 eV, respectively [109]. The biomedical use of Ag_2_X depends on the employed dopant type. For example, Ag_2_Te primarily serves as a biological indicator (fluorophore), but it is rarely applied because of tellurium high toxicity. Silver chalcogenide can be employed for two main applications: high-resolution cellular imaging and in vivo tumor analysis. Jiang et al. [113], Wang et al. [114], and Zhang et al. [115] were the first researchers to report the ability to use Ag_2_S QDs for the in vitro molecular imaging of living cells. Ag_2_S QDs can serve as nanoprobes for cell selection in biomedical imaging applications, as they are effective in NIR II, which explains their high photoluminescence emissions. Wang and Yan [116] reported the usage of Ag_2_S QDs in in vivo imaging, through which it was possible to distinguish cancer cells.

Gold QDs (AuQDs) are ideal particles for biomedical applications because they are non-toxic, inert, and biocompatible. Moreover, they exhibit ordered dispersity, and are easily functionalized. Such features make them preferable for employment in diagnosis, imaging, delivery of medical therapies to cells and tissues, control of surgical procedures, and electromagnetic radiation management [117]. It is worth noting that AuQDs possess the same characteristics as gold nanoparticles (AuNPs) but AuNPs are dissimilar from AuQDs, as they do not fluoresce. AuNPs have SPR-induced colorimetric characteristics which depend on particle size, shape, dielectrical nature, surrounding medium, and surface functionality.

### 5.1. Applications of QDs in Tumor Research

Despite cancer being one of the most-studied diseases worldwide, a comprehensive cure or diagnosis method has not yet been developed. Approximately 40% of the current population suffer from this deadly disease. Scientists across the world struggle to innovate new methodologies and therapies for the treatment of tumors. These techniques usually have a short lifespan due to the dynamic genetic mutations occurring in cancerous cells. As a result, there is a need for innovative technologies to help tackle cancer. Developing appropriate labels are at the main core of bio-labeling investigations. Many conservative techniques already exist which rely on the use of traditional fluorescent labels in tumor research, including organic dyes, which have some drawbacks. These include photo-bleaching effects. As a result, the application of such organic dyes as fluorescent labels is very limited. Semiconductor QDs possess attractive optical features for use in such applications, including broad emission and narrow excitation spectra, extended fluorescence lifetime, and insignificant optical bleaching. The narrow emission spectra and wide range of QD excitation wavelengths means that a single source of light can promptly stimulate different QD particles. The QD emission peaks are symmetrical, narrow, and with minor overlaps. This differs from organic fluorophores′ emission peaks, which are commonly asymmetrical and with wide notable tails. This makes organic fluorophores vulnerable to disruption, so they become difficult to interpret. Furthermore, the emission wavelength of the QDs can be controlled by adjusting their crystal structure and size. QDs also possess a fluorescence intensity that is commonly greater than that of traditional dyes. QD fluorescence intensity and stability are 20–100 times greater than those of organic fluorescent dyes [118]. QDs are potent marker tools for long-term follow-up studies of vital life processes due to their anti-quenching behavior and photo-chemical stability [119]. Moreover, QDs display good optical stability in bio-imaging and a high potential to resist photo-bleaching. The photo-bleaching rate of organic fluorescent dyes makes them prone to fluorescence loss. On the contrary, the rate of photobleaching of QDs is very low [120]. CdSe/ZnS QDs possess a fluorescence intensity for almost 14 h without the signal becoming diminished [121].

As a result of these properties, QDs may become the preferred particle of choice in fluorescence imaging and labeling. Such properties may extend their use into tumor research [26]. The advantageous of photo-physical and -chemical characteristics of QDs make it feasible for QDs to be promising candidates for early diagnosis, prognosis, and monitoring of tumors. Beyond this, QDs have been used in tumor drugs delivery as well as photodynamic therapy (PDT) [122]. Among the various QD applications in tumor research, the imaging of both in vivo and in vitro cells has received worldwide attention [123]. QDs are particularly promising fluorescent labels for tumor cell imaging in vitro because of their distinctive advantages. Modifying the surface coating of QDs enhances their flexibility and labeling efficiency so that they can be used to explicitly and efficiently identify tumor cells at both subcellular and cellular levels. Immune-histochemical (IHC) and trastuzumab-conjugated QD (IHC-QD) techniques were recently developed by Miyashita et al. [124]. Images of the epidermal growth receptors in tumor tissue samples were collected from 37 breast cancer patients. The novel IHC-QD technique could overcome some of the disadvantages of the traditional IHC protocols, such as the auto-fluorescence imaging of tumor cells. Such improvements might enable a more rapid detection of HER2 expression level [124].

In case of in vivo studies, the optical probe fluorescence should effectively penetrate through tumor tissues. NIR molecular probes (700–1000 nm) are more advantageous than visible wavelength-emitting probes. Biological tissues have lower NIR absorption than visible light. This allows NIR light to penetrate deeper into the targeted tissues than visible wavelength light, thereby enabling the assessment of deeper tissues. In addition, at the NIR, there is less autofluorescence than visible wavelengths. As a result, probes emitting light at the NIR region are more suited to and preferred for in vivo imaging. Different QDs have been synthesized with fluorescence emissions ranging from UV to NIR regions, which may be used in imaging of tumor tissues, sentinel lymph nodes, and blood vessels [125]. Compared to many other imaging techniques, like computed tomography and magnetic resonance, QD imaging has the potential to provide further specific information. With respect to tumor imaging *in vivo*, in 2004, Voura et al. [126] developed a technique for tracking tumor cell overexpression within living animal tissues using QD labeling, emission-scanning microscopy, and multi-photon excitation. Gazouli et al. [127] investigated the labeling of vascular endothelial growth factor (VEGF) by the conjugating bevacizumab with QDs. By using this technique, non-destructive imaging of the VEGF-expressing tumor xenografts was successfully achieved in animal models. These studies suggest that QDs might be used as fluorescent trackers to label in vivo tumor cells. In addition, fluorescent indium phosphide (InP) QDs have been modified with anti-VEGF receptor 2 (anti-VEGFR2) monoclonal antibody to develop innovative chemotherapy for tumor cells. MiR-92a inhibitor induced the expression of tumor suppressor p63 [82]. Imaging and treatment of target tumor cells has been shown to be effective using VEGFR2-CD63 and the functionalized InP nanocomposite. For designing heavy metal-free QDs for in vivo imaging, Yaghini et al. [114] synthesized biocompatible QDs with strong quantum photoluminescent output that was suggested for use in mapping of lymph nodes. Furthermore, their low intrinsic toxicity has made them suitable for imaging in vivo tumors.

### 5.2. Applications of QDs in Drug Delivery as Drug Carriers

To date, the roles of QDs in drug delivery are chiefly divided into two main categories, in vivo fluorescent probes and drug carriers and elucidating pharmacodynamics and pharmacokinetics. QDs combined with biological molecules, such as antibodies and peptide ligands, may be used to enhance their targeting and use in drug delivery systems [128]. The first study on the use of QDs for in vivo targeted tumors diagnostic imaging and therapy was published in 2004 [129]. Since then, there has been a number of studies that have used QDs to demonstrate their potential as tools for drug delivery and tumor-targeting therapies [130]. ZnO QDs were developed as an innovative drug delivery system to control the intracellular drug release, such as doxorubicin. Following penetration into cancerous cells, controlled doses of doxorubicin would then be released due to the acidic intracellular conditions [131]. Another novel method using sulfonic-graphene (sulfonic-GQDs) was performed to target in vivo tumor cells [128]. The sulfonic-GQDs penetrated the tumor cells without any bio-ligand modification owing to the high fluid pressure in cancerous tissues. These data suggest that sulfonic-GQDs might be employed as innovative agents in drug delivery.

### 5.3. Applications of QDs in Photodynamic Therapy

At the end of the 1970s, PDT was introduced for the first time as a cancer treatment technique [132]. PDT depends on the use of visible light, and photosensitizers in presence of oxygen molecules. The key theory of PDT is that even after entering the body, photosensitizers appear to accumulate in damaged rather than healthy tissues. At that point, the sensitizing source of light emits the generated photosensitizers, which absorbs the photon energy and transmits it to the oxygen molecules, where photo-oxidation takes place to release reactive oxygen species (ROS). ROS interacts with cells and intracellular macromolecules, such as nucleic acids and proteins, and several subcellular organelles undergo cellular apoptosis or necrosis. Several parameters affect the success of the PDT process, including photosensitizer type and concentration in the targeted cells, laser wavelength irradiation type, duration and intensity and oxygen content of the micro-environment surrounding the targeted cells [133]. Photosensitizers are distinct molecules which can endure photochemical reactions once exposed to light. Developments in photosensitizers have led to the advancement of PDT. Fakayode et al. [134] reported the evolvement of nanoparticle self-lighting PDT (NSLPDT). NSLPDT depends on the use of QD particles, which exhibit a sustained and controlled luminescent light release after excitation. The self-luminescent particles are then conjugated with photosensitizers to synthesize a biological system that can be directly injected into patients with cancer. NSLPDT overcomes the drawbacks of weak penetration of external light sources and the poorly-induced PDT influence, which significantly improves the efficacy of the cancer therapies compared with conventional PDT.

### 5.4. Applications of QDs in Microbial Labeling and Tracking

#### 5.4.1. Single-Virus Labeling and Tracking

Single-virus tracking may provide a clearer understanding of the relationship between viruses and their host cells through the imaging of the infection process, which involves attachment, entry, replication and egress. Up until now, fluorophores usually used for labeling viruses chiefly depend upon organic dyes such as cyanine5, fluorescent proteins such as green fluorescent protein, and QDs. Compared with organic dyes and fluorescent proteins, QDs retain distinctive optical characteristics, for instance, high quantum yield and photo-stability. This makes QDs very suitable for long-term tracking of single-virus with high sensitivity. Depending on the site labeled, QD labeling strategies for virus tracking are often categorized into three major groups: (i) internal components; (ii) external components; and (iii) other components.

QD possible use to identify external viral components was documented through the interaction of biotin-streptavidin with the virus particle (i.e., detected by a primary antibody) linked to a biotinylated secondary antibody and lastly distinguished by streptavidin-conjugated QDs [135]. Joo et al. [136] succeeded in labeling retroviruses with QDs. This took place through a combination between a short acceptor peptide, which was sensitive to attachment with streptavidin-conjugated QDs. The labeling of biotin-streptavidin is an extremely simple labeling technique with improved potency and consistency. However, the labeled viruses appear to have high infectivity. Hence, it is very important to control in situ infection behaviors. To track viral infections, viruses can be labeled with several functionalized QDs by altering the viral surface coating proteins. However, the incorporation of QDs on the viral surface could have an impact on the viral ability to penetrate the host cells, thereby modifying virus-cell interactions, which would make it almost impossible to control late viral infection after losing the viral envelope [137]. Meanwhile, the encapsulation of QDs within the enveloped viral capsids may introduce an ideal solution to tackle these problems. Chemical tracking strategies have been extensively implemented. For instance, adeno virus serotype 2 was labeled successfully using QDs as reported by Joo et al. [138]. The labeling took place via amine-carboxyl crosslinking interaction to strengthen the imaging of the intracellular viral behavior within the targeted living host cells. Zhang et al. [126] suggested the encapsulation of QDs inside the core of Type I human immunodeficiency virus to facilitate viral tracking inside the living host cells. The authors revealed that conjugation of QDs with modified genomic RNAs (gRNAs) containing a viral genome sequence could be incorporated inside viruses. This further allowed the visible tracking of Type I human immunodeficiency virus infection.

Viruses are primarily comprised of DNA/RNA containing their genetic material, a protein envelop (designated as the capsid) that stores viral genetic materials, and, in certain cases, a lipid coat may exist around the protein envelope. Few viruses, however, do not have this complex structure. For instance, a prion is made up of protein rich only with histidine (His), which, in turn, provides an appropriate receptor for binding with divalent metals. Within this perspective, a modified QDs-Ni^2+^ complex of polyethylene glycol-nitrilotriacetic acid was suggested to facilitate labeling of the prion His-rich protein, and tracking the transport behavior pattern [139].

#### 5.4.2. Bacterial Labeling

Accurate and rapid recognition of pathogenic bacteria is of a major biomedical concern [140]. In the last few years, several dyes and fluorescent polymers have been tested for bacterial labeling [141]. However, QDs have also been extensively investigated as promising bioprobe alternatives for bacterial imaging. QDs used for the labeling of bacteria are divided into four main classes; semiconductor QDs, carbon dots (CDs), silicon QDs, and polymer dots (P dots). According to Jones et al. [142], pathogenic bacteria are responsible for a large number of infectious diseases that contribute considerably to the global mortality rate. Among the bacterial species that cause several bacterial diseases are *Clostridium perfringens*, *E. coli* O157:H7, *Salmonella typhimurium*, *Listeria monocytogenes, Mycobacterium tuberculosis, S. aureus*, *Streptococcal* sp., and *Bacillus cereus* [143]. Approximately 2.8 million in the United States of America are infected annually with resistant bacterial strains, which in turn causes almost 35,000 mortalities annually [144]. It is therefore becoming increasingly critical to address effective strategies for tracking and combating pathogenic bacteria in clinical settings, food, and the environment.

Monitoring the number of pathogenic bacteria and, in particular, drug-resistant bacteria is the first and most vital step in controlling their spread. Generally, conventional plate counting and polymerase chain reaction (PCR) are two common methodologies used in bacterial detection and quantification. Although these methods are sensitive and precise, they require specific sample preparation and, as a result, they are time consuming [144]. Consequently, there is a crucial need to enhance the current methods for accelerated bacterial detection. CDs provide promising solutions to such rapid detection methods because of their outstanding fluorescence properties, potent antimicrobial potential, biocompatibility, and biosafety [145]. The efficiency of glowing CDs was proved in bacterial bioimaging and optical detection [146]. Single-walled carbon nanotubes CDs were the first source for isolating CDs. CDs involve quasi-spherical carbon QDs (CDs), graphene QDs (GQDs), and polymer QDs (PQDs) [147]. CDs and GQDs are extensively studied as anti-bacterial agents [148]. They are useful for bacterial detection and imaging as well as determining antibacterial potential. Such applications have been successful due to the interactions between the CDs and bacteria. The presence of functional groups, such as sulfhydryl, amino, hydroxyl, and carboxyl groups on CDs greatly affects their antibacterial potential. This is because of CD charged surface, unique structure, and photocatalytic features which can be easily modified by different functional groups [144]. For instance, amines and amides of *N*-doped-CDs play a vital role in improving the antibacterial effects [149]. The electrostatic interaction occurring between the CDs and pathogenic bacteria might be the main key factor behind CD antibacterial mechanism.

Ampicillin conjugated amino functionalized CDs (CDs-NH_2_) were investigated as a novel approach in antibacterial therapies [150]. The resultant data showed that AMP-CDs generated ROS under visible light irradiation, which were the leading cause behind the growth inhibition of *E. coli*. Most *E. coli* strains are commensal and harmless, although there are also some pathogenic strains. Examples of such bacterial sub-strains include *E. coli* O157:H7 and O104:H4. Both strains are related to recent outbreaks in EU countries (Germany in 2011 and the UK in 2005 and 2009) and North America (1996 and 2006) [151]. Effective, precise, and rapid detection of *E. coli* is generally desired. Rapid detection of such bacteria has become a priority in a range of different disciplines, including those relating to food, water, and the environment [152]. There are a number of common methods for the detection of *E. coli*, including the membrane filtration, most probable number, and chromogenic enzyme-substrate techniques. Nevertheless, these techniques are either labor- or time-intensive [151]. Therefore, Yang et al. [151] documented a novel and potent approach for *E. coli* cell labeling with QDs. Two types of QDs were used; CdTe and CdTe/ZnS. Both QD types were used because they exhibit good biocompatibility with emission wavelengths near to the flow cytometric excitonic absorption wavelength. Hence, enabling rapid and accurate detection. Permeability was analyzed using SEM and activity measurements of the released alkaline phosphatase (PhoA) from periplasm. This method has opened a new avenue for the facile, quick, and sensitive detection of bacteria.

#### 5.4.3. Fungal Labeling

Around 400 fungal species are identified as human pathogens, 50 of which cause several neurological disorders [153]. For instance, *F. oxysporum* is an opportunistic fungus, which becomes deadly to immunocompromised patients. It is therefore important to establish new and highly sensitive approaches for the early fungal detection. PCR can be used to detect fungi. However, PCR techniques are expensive and need a significant quantity of fungal biomass [154]. Additionally, because of the distinctive optical characteristics of semiconductor QDs and CDs, in recent years, their suitability for use in fungal labeling has been noted. Some *Candida* species are associated with superficial or invasive infections to humans, particularly in immune-depressed patients [155]. *Candida parapsilosis*, *C. tropicalis*, *C. krusei*, *C. glabrata,* and *C. albicans.* are among the most common *Candida* species responsible for candidiasis. The biochemical components of the cell wall in *Candida* spp. are not only responsible for their cellular integrity but also for the interaction between *Candida* sp. and the environment. The cell wall is involved in biofilm formation and interactions with the host cells [156]. Hence, any changes in the polysaccharide structural composition of the cell wall will directly affect the pathogen-associated molecular patterns [157].

### 5.5. MicroRNAs (miRNA) Detection

miRNAs are a category of non-coding small nucleotide RNAs and are approximately ~22–23 nucleotides long. They act as monitoring molecules in RNA silencing and are included in almost all pathological and developmental pathways in animals and humans [158]. After miRNAs were first discovered in *Caenorhabditis elegans* [159], they were subsequently identified in all living organisms and even some viruses. Nevertheless, miRNAs were not recognized as a biological regulator class until the early 2000s. Since then, more than 30,000 miRNAs have been discovered [160]. Deregulation of miRNAs can cause some illnesses, such as cancer or cardiovascular disorders [161]. Up or down alteration of miRNA expression can have deleterious consequences on cellular processes, for instance, proliferation and apoptosis. In this context, miRNAs introduce a rich biomarker base for the recognition of various illnesses.

The analysis of miRNAs is more challenging than that of other oligonucleotide targets for a number of reasons, including sequence homology among family members, short coding length, vulnerability to decomposition, and limited availability in RNA samples. Various techniques have been innovated to meet the criteria required for miRNA analysis. The identification theory is dependent upon Watson-Crick base-pairing and base-stacking. In situ hybridization, real-time PCR, Northern blotting, and various microarrays are among the most commonly used techniques for detecting miRNAs. The miRNA regeneration and reduced stability, time-consuming nature of the mentioned methodologies, and the need for separation procedures are the key drawbacks for their widespread use [162]. New techniques for the simultaneous detection of single and multiple short RNAs are being actively pursued for elucidating gene expression profiles in biological systems and for the early diagnosis of cancer and other illnesses. To increase the sensitivity, a number of new procedures have been proposed, including the nucleic acid-based locked probing assay, the ligation chain size-coded reaction, and probe-based exponential circle rolling amplification. Chen et al. [163] introduced a new electro-chemiluminescent (ECL) biosensor for detecting miRNA via the interaction of QDs conjugated with doxorubicin (DOX-QDs) with the DNA/RNA hybrids. This interaction caused the subsequent amplification of ECL emissions. The elevated ECL strength was shown to be directly correlated to the quantity of targeted miRNA in the tested samples.

## 6. Conclusions, Challenges and Future Prospects

Research on QDs has drawn worldwide attention from the scientific community. Growing knowledge of green chemistry principles and biological processes has led to the establishment of environmentally sustainable approaches to synthesize non-toxic and eco-friendly QDs. Unlike most of the chemical and physical QD synthesis techniques, which involve noxious chemicals and costly energy requirements, microbial synthesis of QDs is cost-effective and eco-friendly, and does not require any external reducing, capping or stabilizing agents. Microbial synthesis of QDs has therefore emerged as an attractive branch of nanobiotechnology. Furthermore, due to the high microbial diversity which mediates the fabrication of QDs, microbes are considered as potential biological factories.

This review provides a comprehensive description of the basics of QD science since their early discovery, their structural composition, their distinctive features, and the synthesis of QDs using microbial machinery, such as bacteria, yeast, and fungi. Analysis of microbially-fabricated chalcogenide QDs is discussed with regards to their structural and optical features. The structural properties have been determined in context with XRD, FTIR, SEM, and TEM analyses. Optical characterization of QDs was demonstrated via UV/Visible and fluorescence emission spectra, providing insight into the arrangements of energy levels inside the particles. In addition, the versatile biomedical applications of QDs and emerging obstacles are discussed. Despite their successful results, certain anticipated problems, such as toxicity, have also been observed; thus, the use of QDs remains controversial. Capping QDs with functional materials may result in complex lethal immune reactions. Additionally, heavy metals present in the core may be toxic to host cells. For the full determination of the cytotoxicity of QDs, an integration between the in vitro and in vivo investigations is mandatory. Toxic effects of metal chalcogenide QDs are particularly dependent on several factors including QD sizes, surface configurations, exposure routes, and agglomeration of the prepared particles. Extensive studies are therefore required to elucidate the relationship between the toxicity and the factors listed above.

The use of microbes to mediate the synthesis of QDs is remarkably significant; therefore, further studies are required for commercial development. Certain limits restrict the commercial production and applications of QDs, including: (i) the relative increasing in QD sizes during synthesis; (ii) controlling the synthesis stages such as nucleation, crystallization and crystal growth; and (iii) boosting the synthesis rate of QDs by optimizing the variables affecting the synthesis process and the downstream processing methodologies.

Future research should be directed toward: (i) identification of the exact mechanism behind the microbial synthesis of QDs; (ii) elucidation of the enzymes playing a key role during the nucleation and growth of QDs; (iii) exploration of the distribution and mode of action of QDs to promote their biomedical applications; (iv) determination of the effects of the exposure to low doses of QDs for a long time; and (v) assessment of risk management arising from QD preparation, handling, and storage procedures.

## Figures and Tables

**Table 1:** List of microorganisms (bacteria, yeast, and fungi) mediating quantum dots (QD) production and different types of microbially-produced QDs.

Microorganisms	QDs	Factors Optimization	References
**Bacteria**			
*Desulfovibrio desulfuricans* NCIMB 8307	ZnS	-	[12]
Genetically engineered *Escherichia coli*	CdS	Reactant concentrations, reaction time	[73]
*Stenotrophomonas maltophilia*	CdS	Reaction time	[74]
*Clostridiaceae* sp.	ZnS	-	[75]
*Acidithiobacillus ferrooxidans, A. thiooxidans* and *A. caldus*	CdS	pH	[76]
*Pseudomonas putida* KT2440	CdS	CdSO_4_ concentration and exposure time	[77]
*E. coli BW25113*	CdS	-	[78]
*E. coli*	CdS	Reaction time	[79]
*E. coli*	CdTe	-	[80]
*P. chlororaphis* CHR05	CdS	CdSO_4_ concentration, temperature, time and pH	[81]
**Yeast**			
*Saccharomyces cerevisiae*	CdS	-	[82]
*S. cerevisiae* MTCC 2918	ZnS	Reaction time and different concentrations of yeast biomass and ZnSO_4_	[83]
*S. cerevisiae*	CdSe	Effect of *S. cerevisiae* growth phase, selenite concentration, cadmium concentration, effects of selenite and cadmium incubating time	[84]
*Schizosaccharomyces pombe*	CdS	-	[85]
*Rhodotorula mucilaginosa*	CdSe	Different concentrations of Na_2_SeO_3_ and CdCl_2_ and pH	[86]
**Fungi**			
*Fusarium oxysporum*	CdTe	-	[87]
*Phanerochaete chrysosporium*	CdS	-	[88]
*F. oxysporum f.* sp. *lycopersici*	CdS	Reaction time	[89]
*Rhizopus stolonifera*	CdTe and CdS	-	[90]
*Pleurotus ostreatus*	CdS	-	[91]
*Aspergillus terreus*	PbSe	-	[92]
*Aspergillus* sp.	ZnS	Reaction time, temperature, pH	[93]
*Penicillium* sp.	ZnS	-	[94]
*Trametes versicolor*	CdS	-	[95]
